# Barriers and enablers to a balanced diet among UK medical students on clinical placement: A qualitative study

**DOI:** 10.1177/02601060251400243

**Published:** 2025-12-15

**Authors:** Maria VM Karadimova-Watts, Niki Jakeways

**Affiliations:** 1 405987 GKT School of Medical Education, King's College London, London, UK

**Keywords:** Medical student diet, higher education, university, dietary barriers, dietary enablers, university support

## Abstract

**Background:**

How universities support medical students to attain balanced diets represents an important area to help students improve their physical and mental health, alongside their academic performance. Although dietary barriers have been studied in the general population, they have been minimally studied in medical students; a group expected to give dietary advice in their future career.

**Methods:**

Nine semi-structured interviews were conducted with fourth- and fifth-year medical students at a London university. Participants were asked about their perceived dietary barriers, enablers and strategies to tackle barriers and promote enablers. Transcripts were thematically analysed.

**Results:**

Three superordinate themes were derived from the data: proximity to food options and support network, expenditure of limited resources and prioritising health and knowledge. Key enablers were social eating and feeling well-nourished, while key barriers were inaccessibility, increased time and costs. Some influences were both enablers and barriers. For example, peer influence: positive in instances of good cooking skills, but negative in instances of pressuring to eat out regularly. Additionally, participants suggested both personal and university-based strategies. Some personal strategies included splitting food costs and preparation amongst the household, whereas some university strategies comprised improving the variety and convenience of healthy food on campus.

**Conclusion:**

Moving forward, the student-suggested strategies have the potential to improve the operational aspect of university-based dietary support; for example, including formal dietary advice and guidance in induction lectures. Through having a greater focus on student diet and nutrition, universities may be able to better nurture academic success, physical and mental health.

## Introduction

Unhealthy diets are a major health concern, contributing to millions of deaths worldwide (Afshin et al., 2019). Typically, such diets are high in sugar-sweetened beverages and processed red meats, and are low in whole grains, fruits, vegetables, nuts and legumes (Afshin et al., 2019; Cena and Calder, 2020). In contrast, a balanced diet, as reflected in the UK Government's *Eatwell Guide* (British Nutrition Foundation, n.d.), has been found to improve morbidity, mortality and academic performance (Antonopoulou et al., 2020; Scarborough et al., 2012). Given increasing concerns about the nation's eating habits, it is pertinent to explore medical students’ attitudes towards balanced diets, as these have implications for both their future health and the guidance they provide to patients.

In the UK, despite 75% of consumers claiming to understand healthy diets (Food Standards Agency, 2021), only 0.1% of the population adhere to the *Eatwell Guide* (Scheelbeek et al., 2020). This demonstrates that dietary behaviours require navigation of multiple influences and are shaped by complex factors, including knowledge of healthy eating and cooking skills. This links with the concept of food literacy, defined as ‘the ability of an individual to understand food in a way that they develop a positive relationship with it, including food skills and practices across the lifespan in order to navigate, engage, and participate within a complex food system’ (Cullen et al., 2015: 143). Poor food literacy is a major contributor to diet-related mortality (Silva et al., 2023), and hence, improving food literacy is essential to reduce diet-associated deaths.

Food literacy is particularly important for young adults, including university students, as this is when lifelong habits such as diet are established (Parcel et al., 1988: 41–42). Understanding the influences shaping university students’ dietary perceptions offers opportunity for early intervention. Via a questionnaire study, Sprake et al. (2018) identified that universities could improve student diets by increasing nutrition skills through engaging students in food preparation. However, their model of dietary patterns did not account for social or cultural influences, highlighting a knowledge gap amongst medical students. Capturing medical students’ experiences of dietary influences is crucial, as they soon become doctors with responsibility for dietary counselling. Alongside improving dietary understanding, a balanced diet (such as the Mediterranean diet) has been associated with higher academic performance (Antonopoulou et al., 2020), and culinary education has been shown to improve readiness for dietary counselling (Wood et al., 2021). Thus, a medical student's dietary understanding is important for their professional and academic careers.

As medical students transition into clinical practice, it is also important to consider doctors’ diets. Current evidence suggests that doctors’ diets are suboptimal: in Ireland, alcohol consumption has been found to be above average (O’Keeffe et al., 2019); in Nigeria, only 25.4% consume fruit daily (Ambakederemo and Chikezie, 2018). Similar trends of poor diet appear among medical students – 38% of Arabian medical students were found to be overweight or obese, mirroring the global adult average of 43% (Ahmed et al., 2019; WHO, 2024). Given the potential stressful demands of clinical placements, the bi-directional relationship linking diet and stress should be considered (Bremner et al., 2020). For example, 11.2% of American medical students responding to a questionnaire described their stress levels as severe and debilitating (Hill et al., 2018); notably, clinical placements are thought to contribute a significant emotional challenge (Weurlander et al., 2018). Therefore, exploring the dietary influences on clinically-placed medical students has important implications for both physical and mental health.

Most existing studies have explored medical students’ baseline diets globally (Glore et al., 2013), with some exploring dietary barriers outside of the UK (Ahmed et al., 2019; Bergeron et al., 2017). Among Californian medical and pharmacy students, Bergeron et al. (2017) identified lack of time, high food costs, reduced accessibility to healthy food and poor nutritional knowledge as major barriers. Similarly, Ahmed et al. (2019) found that Bahraini medical students perceived fast foods as more appealing, and were negatively influenced by social networks. UK-specific research is limited; however, via 15 semi-structured interviews with students at a London university, Aydin (2025) suggests that students were well informed regarding healthy diets, but were primarily influenced by financial cost, limited availability and variety of healthy food options. Although not specific to medical students, this study offers a foundational knowledge of dietary influences among UK university students.

Therefore, there is scope to explore the dietary influences affecting UK medical students in further depth, as current research is largely international, includes non-medical cohorts, or focusses mainly on dietary content. This demonstrates a missed opportunity for universities to deliver dietary support. Nutrition-focussed university policies could enhance medical students’ academic performance, improve their mental and physical health, and readiness for dietary counselling.

Therefore, this study aimed to explore the following research questions:

1. What barriers and enablers do UK medical students experience in achieving a balanced diet, while undertaking clinical placements?
2. What ideas, suggestions and strategies do UK medical students have to reduce barriers and encourage enablers, while undertaking clinical placements?

## Methods

As students have varying and subjective experiences of their diets, this topic was explored within an interpretative paradigm, which acknowledges that individuals subjectively experience multiple realities and construct knowledge differently (Simmons, 1995). Semi-structured interviews were chosen to facilitate discussion and exploration of participants’ views (Britten, 1995).

### Participant recruitment

Inclusion criteria were being a fourth- or fifth-year medical student at the university. Recruitment took place via the university newsletter and medical student fourth- and fifth-year WhatsApp group chats. As voluntary response sampling was unsuccessful, convenience sampling was used; consequently the primary researcher knew some participants professionally and personally. Participants were assured of confidentiality, and no incentives were issued. Interview questions were not shared in advance, although an information sheet outlining the study's aims was. Participants were informed of the researcher's stage of training, interest in nutrition and medical education, and that the project formed part of her dissertation.

### Interviews

Nine semi-structured interviews were conducted from January to February 2024, by the primary researcher, with no participant dropouts. Interviews averaged 23 minutes, allowing ample participant-led discussion on dietary influences and improvement strategies. The online interviews involved the researcher and participant only, with recording and transcription taking place on Microsoft Teams. Anonymised transcripts were securely stored on the university OneDrive and were not returned for participant review. Limited demographic data (gender, year, accommodation type) was collected to contextualise responses. Further demographic data was not collected, as the study aimed to generate insights rather than represent all medical students. Field noting and open questioning facilitated discussion of medical school experiences, placements, current diet views, dietary barriers, enablers and strategies.

The number of interviews was guided by prospective participant interest in the study and data saturation. Codes were generated contemporaneously as the interviews took place, and remained consistent during analysis. Interviews continued until similar codes repeatedly emerged, and no new codes were emerging, which was felt to be data saturation (Braun and Clarke, 2021). Furthermore, no interviews were piloted, repeated or removed. See the interview guide in the online Supplemental materials.

### Data analysis

An inductive approach was used to generate findings from the raw data (Simmons, 1995; Thomas, 2006). The researcher conducted reflexive thematic analysis (Braun and Clarke, 2006, 2019), on anonymised transcripts, assisted by NVivo Version 14. Analysis followed Braun and Clarke's six steps of thematic analysis: data familiarisation, initial coding, theme development, review, definition and reporting. This process remained flexible and reflexive, through continual questioning and theming of the data throughout all stages (Braun and Clarke, 2019).

This approach acknowledged that themes are subjectively interpreted by the researcher, rather than representing a single truth (Braun and Clarke, 2019). Reflexivity was maintained through journaling, supervisor discussions and reflecting on underpinning assumptions as an insider researcher (Braun and Clarke, 2019; Mercer, 2007). To mitigate potential biases, all codes and themes were reviewed with the supervisor, and any differences in interpretation were discussed, ensuring alternative perspectives informed final interpretations. The researcher derived themes from the data during February to April 2024. Due to time constraints, member checking was not conducted.

### Ethical approval

Minimal risk ethical approval was provided by the King's College London Ethical Committee (MRSU-23/24-40732) on 20/12/23. All participants gave electronic consent to take part in the study and for their direct quotations to be used in the manuscript.

## Results

Of the nine participants, seven were female and two were male; eight were in their fourth year, and one in their fifth year. The participants were given student numbers (S) for identity.

Three superordinate themes of influences on balanced diet achievement were identified (Table 1), spanning dietary barriers, enablers and strategies.

**Table 1.** Superordinate and subthemes of influencing factors for achieving a balanced diet.

Proximity to support network and food options as an influence on diet
Food as a means of social connection Accessibility and convenience of food options
Expenditure of limited resources as an influence on diet
Increased time for planning, preparing and consuming healthier food The perception that healthier food is more expensive, unhealthier food is cheaper
Prioritisation of health and knowledge as an influence on diet
Participants’ health status and its influence on prioritising food Nutritional knowledge and skill levels informing food choices Beliefs and attitudes informing food choices

### Proximity to support network and food options as an influence on diet

Six participants described their diet as rooted in their social network (S1, S3, S4, S5, S7, S9). Shared meals with flatmates enabled participants: ‘it holds me accountable to make sure I'm eating my three meals’ (S4), suggesting that eating alone reduced motivation to maintain regular mealtimes. Conversely, the absence of a support network was a barrier, demonstrating that meals were not seen only as nutritional intake, but rather an opportunity for social connection. One student described that ‘placement can be quite isolating… You're literally the only one who's free to have lunch at that particular time’ (S8). Limited support also increased the burden of food preparation (S5), whereas living at home enabled healthier eating through homemade meals (S1, S8). Overall, students perceived shared meals as beneficial for both preparation and consumption of food.

Friends acted as both enablers and barriers. Good cooks were ‘inspiring’ (S7), but social pressures to eat out and drink alcohol often hindered healthier choices (S2, S8). Eating out also risked dietary needs being overlooked (S1). These views demonstrate variability in the peer influence, and how the nature of certain friendships can reflect the dietary choices a student ultimately ends up making.

Beyond social circles, familial influences and upbringing also shaped food habits, illustrating the bi-directional influence of immediate environment on dietary perceptions. Some participants described inheriting ‘good habits from home’ (S5). For others, cultural traditions encouraged fewer balanced foods: ‘Pakistani culture… curries, oily food, chapatis… heavy carbohydrates’ (S9).

Aside from upbringing, convenience at clinical placement prevailed as a dietary barrier (S5, S6, S8). Participants reported limited access to healthy options: ‘If I had the convenience of making healthier choices at places where I’m going to buy food anyway, that would help’ (S6). Other participants criticised the irony of fast food in hospitals (S7, S8): ‘We’re meant to advocate for health, but we’re fighting a losing battle here’ (S7). These aspects highlight how medical students may often be short for time, and in need of a convenient option, although are disadvantaged by the options available.

To improve accessibility and convenience of healthy options, students proposed practical solutions, including free food (such as fruit) at placements (S1, S2, S3, S5, S7). Additionally, as limited access to kitchenettes on placement was reported as a barrier (S1, S3, S4, S5), students suggested that enabling access would reduce reliance on unhealthier foods (S1, S3, S4), such as sandwiches (S4). This was particularly relevant for those changing placement sites or using temporary accommodation (S3, S5). Additionally for those commuting to placement, transporting food was seen as impractical: ‘What do I bring on this two-hour commute?’ (S1). Overall, access and convenience strongly shaped participants’ dietary choices.

Outside of placement, two participants identified limited access to organic food shops as a barrier (S1, S8). Although, enablers comprised family members purchasing groceries (S8), and owning a car to improve the accessibility of shops (S3). Thus, convenience extended beyond placements to encompass broader food accessibility at home.

### Expenditure of limited resources as an influence on diet

All participants associated healthier foods with higher demands on time, including planning, shopping, cooking and eating meals. Planning meals was particularly challenging when personal study took priority: ‘you don't really wanna spend that energy thinking “what meals should I be making?”’ (S2). Additionally, grocery shopping was time-consuming (S3, S4, S5, S7, S8), and without preparation, students risked skipping meals: ‘I sometimes end up going through the week living off water and air’ (S7).

Cooking (S1, S3, S4, S5, S6, S9) and eating (S1, S4, S7, S8, S9) were similarly time-intensive, especially alongside clinical placements. However, most students acknowledged ‘meal-prepping’ as a time-saving strategy (S1, S2, S3, S4, S6, S7, S8, S9), and some suggested ‘splitting the load’ (S4) with members of their support network (S3, S4). This demonstrates that the time burden of clinical placements alongside personal study often left students with little remaining time, thus negatively influencing their food choices.

Placement schedules shaped eating patterns and therefore dietary choices, although with contrasting effects. While one participant described a regular 9.00–17.00 schedule which enabled reliable lunch and dinner times (S1), others frequently missed meals due to back-to-back sessions (S1, S3, S4, S7, S9): ‘I’ve got to either eat really fast or eat in the teaching session… Or I just have to miss lunch altogether and I’ll just eat it on the train home’ (S1). Participants suggested introducing mandatory protected lunch breaks (S7, S9). This highlights timetable variability across placement sites, indicating that dietary barriers and enablers can shift between clinical placement rotations.

Cost was widely reported as a barrier, with all participants viewing healthier food as more expensive, including at clinical placements (S1, S8). Additionally, five participants felt that being a medical student was a barrier to achieving a balanced diet (S1, S3, S5, S7, S9). This was particularly due to limited student funding available: ‘I think a student on NHS bursary, student finance and a bit of part time work, which mind you, you wouldn't have time to do because of how heavy clinical placements are in the last two years… . It's almost impossible’ (S9). This demonstrates that although medical students often have the desire to eat healthier, they are disadvantaged by the cost of healthier food options.

In contrast, one participant felt enabled as she could afford her desired food options (S1). However, on the whole, participants suggested that the university could improve dietary-based financial support (S2, S3, S4, S5, S6, S8). Participants also suggested personal strategies, such as seeking more deals (S1, S4, S5), home cooking more often (S2), and splitting the cost of groceries with flatmates (S4). These strategies highlight that dietary barriers and enablers are multi-faceted, and that small actions may meaningfully improve access to healthier food options.

### Prioritisation of health and knowledge as an influence on diet

Seven participants prioritised their health which enabled balanced diet achievement (S1, S2, S4, S6, S7, S8, S9), though others struggled to balance it with competing demands at university (S5). Personal circumstances and experiences influenced diet prioritisation. For example, having no health ailments or allergies enabled good diet through reduced dietary constraints (S1). Although, having experiences of poor health could also motivate healthier choices: ‘he [dad] had gastric cancer… so we have always had to eat quite well’ (S5). To target prioritisation skills, three participants suggested working on their ‘self-discipline’ (S6), alongside the university improving the variety of healthy foods offered to make it easier to prioritise healthier options (S2, S6, S8).

Dietary knowledge appeared to underpin attitudes towards food. Seven participants explained that a key enabler was understanding the importance of food as sustenance for their body (S1, S4, S5, S6, S7, S8, S9). Additionally, several participants perceived their medical education as advantageous in enhancing their dietary knowledge, with two describing themselves as advantaged by their training (S2, S6) and two expressing mixed views (S4, S8). One participant reflected that ‘seeing patients with cardiovascular disease’ heightened their understanding of dietary impact beyond that of the general population (S2). Yet some argued that nutrition teaching was inadequate (S1, S7), and five participants proposed that universities should promote healthy eating among students (S1, S4, S6, S7, S9).

In contrast, two participants maintained that health knowledge is determined by individual priorities, suggesting that students should utilise available resources rather than rely on university teaching (S6, S9). Despite awareness of the implications of poor diet, skills gaps persisted: two participants reported limited prior reflection on their dietary habits (S6, S7), while another lacked confidence preparing certain foods, particularly meat (S7). These accounts highlight the complex interplay between knowledge, prioritisation and perceived responsibility in shaping dietary influences.

Attitudes were complex and nuanced influences on diet. For example, ‘you only live once’ (S6) prompted impulsive indulgence but also reinforced long-term healthy choices. Enjoyment of food (S1, S4, S7, S9) and autonomy in choices (S1, S2, S4, S6, S8) were enabling, whereas distrust of others’ cooking (S8) and associating guilt with specific foods (S8, S9) were barriers. Attitudes often influenced dietary variety, which was valued as an enabler to reduce repetitiveness (S2, S4, S5, S6, S8). However, lack of variety was a barrier due to overfamiliarity (S2, S3, S4, S5), even if it saved thinking time (S2, S4, S5). Personal strategies to improve variety included ‘adding’ to meals (S3), keeping the kitchen stocked (S8), and cooking with others for meal inspiration (S7). These findings illustrate a bi-directional relationship between attitudes and dietary choices, showing that influences can be both internal (personal attitudes) and external (availability and variety of options).

A summary of personal and university-based strategies is presented in Table 2.

**Table 2.** A summary of the personal and university-based suggested strategies derived from the interviews.

Superordinate theme	Subtheme of strategy	Personal strategies	University strategies
Proximity to support network and food options as an influence on diet	Social-connection-promoting strategies	N/A	N/A
	Accessibility- and convenience-promoting strategies	N/A	Ensure equal access to kitchenette spaces (at hospitals and free accommodation) Support free-food initiatives at hospitals Improve convenience of healthier food options on campus or hospital sites
Expenditure of limited resources as an influence on diet	Time-saving strategies	‘Meal prep’ (i.e., bulk prepare meals in advance) Plan meals in advance Set aside time to cook in one's schedule Split up the cooking with members of the household	Ensure designated lunch breaks while on placement Distribute timetables earlier to allow students to better plan their schedules
	Cost-promoting strategies	Deal- and reduced-focussed shopping Home cook rather than eating out Reduce food waste and the need to purchase new produce Split the cost of groceries with members of the household	Create food-related funding opportunities for student societies Create food hardship funds for students Support free-food initiatives at hospitals Encourage student discounts in canteens at hospital sites Supply food vouchers for students Incentivise purchasing of fruits on campus Make the healthier food options offered at university venues cheaper
Prioritisation of health and knowledge as an influence on diet	Health-promoting strategies	Improve self-discipline when adhering to a balanced diet Improve mindfulness and self-awareness when eating	Improve convenience of healthier food options on campus or hospital sites
	Knowledge-promoting strategies	Take ownership for one's own knowledge about nutrition	Advertise nutritional content on food packets Educate students on food options nearby hospital sites Further student teaching on nutrition counselling for patients Educational healthy eating posters for students at hospitals Educate students on eating well as a student
	Attitudinal strategies (variety-promoting strategies)^a^	‘Add’ more to meals to increase the variety of food groups Mindfully choose where to eat out, in advance of eating out Engage with other cooks for new recipes Keep a fully stocked kitchen	Improve the variety of food offered at university food venues

These strategies can guide future learning on improving dietary support for students.

^a^ Participants identified that variety-promoting strategies would improve their attitude towards food, thus, as a form of attitudinal strategies, variety-promoting strategies have been suggested.

## Discussion

### Exploring perceived barriers and enablers to achieving a balanced diet

Key enablers included sharing meals with others and valuing food for well-being, while key barriers were lack of time, inconvenience and affordability of healthy food. Medical knowledge acted as both a barrier and an enabler: some participants felt under-informed about nutrition, while others believed their medical education increased awareness of dietary disease.

These findings suggest that while medical students recognise the importance of a balanced diet, the structural and psychosocial determinants often outweigh the effects of their awareness. Marquis (2005) echoes this, through finding that in Canadian university students (of which 14% were medical students) that food convenience is most important. Additionally, findings consistent with broader university populations were increased time commitments, expensiveness of healthy food (Abdelhafez et al., 2020; Irwin et al., 2019) and negative social influences (Hilger et al., 2017).

Interestingly, through a student-wide questionnaire, Bjorksten et al. (1983) suggested that medical students experienced the same problems as other students, but ‘complained’ more intensely. This may reflect additional stressors seen in medical students, such as high rates of depression (Rotenstein et al., 2016), medical school culture of feeling guilty if not studying during free time (Bergmann et al., 2019), and high levels of maladaptive perfectionist personality traits (Eley et al., 2020). Together, these factors likely magnify negative perceptions and limit coping capacity.

Overall, the findings imply that time, cost and social factors are not unique to medical students, but the intensified stressors may heighten their impact. Addressing mental well-being and workload may therefore be as crucial as improving dietary access or affordability.

### Exploring ideas to reduce barriers and encourage enablers

Participants proposed both personal and university-driven strategies (Table 2). Personal approaches such as meal planning and preparation were viewed as practical ways to save time and diversify diet, while university-level suggestions focussed on improving access to affordable, healthy food (such as free fruit or healthier campus options). Opinions were divided on responsibility for nutrition education: some viewed it as individual responsibility, others as part of medical training.

The emphasis on meal preparation aligns with evidence that structured meal planning supports healthy eating (Ducrot et al., 2017; Fernandez et al., 2020). Similarly, institutional initiatives to increase healthy food availability may improve dietary accessibility (Hilger-Kolb and Diehl, 2019). However, Hilger-Kolb and Diehl's study population were university students based primarily on campus, as opposed to medical students moving between hospital placements, potentially explaining why our study revealed more diverse strategies (personal and university-based).

Nevertheless, the aforementioned importance of convenience (Marquis, 2005) could explain why participants regularly suggested improved access, affordability and provision of healthy food options. In contrast to participant perception, Van Den Bogerd et al. (2020) found no difference in fruit or vegetable intake, following intervention of free fruit and vegetables at a Dutch university campus. However, the study's findings imply that deeper behavioural factors and existing habits may play a more decisive role in determining dietary choices than initially considered.

Therefore, an integrated approach should be sought that targets the physical barriers alongside the cognitive and emotional factors which shape medical students’ eating behaviours. In practice, this means that universities should combine environmental interventions (such as free fruit) with behavioural (by improving convenience) and educational (such as signposting) components to achieve sustained dietary change and minimise dietary barriers.

### Strengths and limitations

The data was collected, coded and themed by a single researcher. Although credibility may have been reduced by the absence of multiple coders (Noble and Heale, 2019), trustworthiness was promoted through a critical self-reflexive journal of internal dialogue and assumptions (Nowell et al., 2017; Tracy, 2010). As described by Braun and Clarke (2023), the researcher's introspection is central to identifying themes, thus, the beneficial reflexive insider stance of the researcher may have helped to elicit more insightful information.

A limitation includes the use of convenience sampling, although the primary researcher was unaware of the participant's dietary views prior to recruitment. Critically, the convenience sampling may have introduced a social desirability bias in the participants (Grimm, 2010), potentially amplified by the professional and personal relationships between the researcher and participants. As described by Kim (2023), power dynamics are common in interviews, although acknowledging their influence on the interview can enhance research depth. In order to reduce the influence of power dynamics and the risk of potential social desirability and confirmation biases, the primary researcher opted for semi-structured interviews. These interviews encouraged open responses while allowing participants to guide the discussion. Although, all participants were at a higher stage of medical training compared to the primary researcher, reducing the risk of hierarchical power imbalance.

Additional limitations include the absence of member checking, the focus on only fourth- and fifth-year medical students at one university and the limited demographic data collected. These factors could potentially reduce the generalisability of the study's findings.

### Implications

This is the first study to explore barriers, enablers and strategies for balanced diet achievement, among UK medical students on clinical placement. These results build on existing knowledge of dietary influences affecting the general UK university student population which are thought to be enabled by good health knowledge, but restricted by barriers such as financial limitations, limited healthy food options on campus, irregular meal patterns, time restrictions for meal planning alongside stress levels (Aydin, 2025). While the findings of this study largely mirror some of the key factors identified by Aydin, we note that opportunities for social connection, student's general health, knowledge, skills, beliefs and attitudes are also key dietary influences. These findings highlight the importance of a holistic approach to supporting medical students’ diets, with student-suggested strategies offering guidance for universities. Crucially, improving dietary support for medical students may improve mental and physical health, as well as academic performance.

### Recommendations

This study involved medical students from one university, thus future studies could explore attitudes from students at different universities and consider the demographical and cultural factors which may influence medical student perceptions. Using qualitative and quantitative questionnaires could also reach more students, providing broader insights to guide university support and improve generalisability. Furthermore, future studies could evaluate and pilot the strategies proposed in this study, to assess their feasibility, cost effectiveness and impact.

## Conclusion

The findings of this study extend existing knowledge that influences on medical students’ diets are complex. Proximity to social networks and the desire to eat for nourishment were prevalent enablers, while the perception of increased time, costs and inconvenience associated with healthy foods were prevalent barriers. Students prioritised health, and the role of food and nutrition in this, to varying extents. Several strategies were suggested to promote a balanced diet, ranging from bulk meal preparation and planning, taking initiative to educate oneself on nutrition, to the university ensuring regular lunch breaks, providing free food initiatives on placements and increasing the variety of affordable, healthy options available.

Student-suggested strategies provide some guidance how to improve student support policies, to become more nutrition-focussed, which could be investigated via pilot intervention studies. For example, introducing new policies to financially support students to access healthier food options (such as food vouchers or hardship funds), alongside introducing nutritional support into student services (such as training student support advisors on nutrition and delivering dietary advice guidance during induction lectures). Through supporting student diet, universities may nurture the development of a healthier medical workforce who are better equipped to share their knowledge and role-model healthy eating habits to patients.

## Supplemental Material

sj-docx-1-nah-10.1177_02601060251400243 - Supplemental material for Barriers and enablers to a balanced diet among UK medical students on clinical placement: A qualitative study Supplemental material, sj-docx-1-nah-10.1177_02601060251400243 for Barriers and enablers to a balanced diet among UK medical students on clinical placement: A qualitative study by Maria VM Karadimova-Watts and Niki Jakeways in Nutrition and Health

## Data Availability

The interview transcripts are available upon contacting the author at the above email address.
