# Supplementary material for: Barriers and enablers to a balanced diet among UK medical students on clinical placement: A qualitative study
Source: Nutr Health. 2025 Dec 15;32(6):1941–50. doi: 10.1177/02601060251400243 (PMC13744587; doi:10.1177/02601060251400243)
Supplement: Supplementary file 1 [file sj-docx-1-nah-10.1177_02601060251400243.docx]

# **Supplementary File- Interview Guide**

**INTERVIEW GUIDE: Exploring barriers and enablers to achieving a balanced diet in UK medical students- A Qualitative Study**

**Contents**

Section 1- Introduction (Prior to transcription)

Section 2- Demographical Characteristics

Section 3- Baseline Diet

Section 4- Barriers to diet & methods to reduce these.

General follow up questions.

Section 5- Conclusion

**Section 1- Introduction (Prior to transcription)**

Thank you for agreeing to participate in my study on the barriers to achieve a balanced diet in Medical Students.

Have you had a chance to read the information for participants and complete the consenting questionnaire? This interview will take roughly 30 minutes and the results will be anonymised.

The data generated will be used for a dissertation project, with hope that these findings will be taken to the Medical School to see whether further support can be put in place.

Are you still happy to participate in this interview? You are entitled to stop the interview whenever you would like.

Do I have your consent to begin the transcription and recording? Do you give consent to use pseudonymised quotations and responses from the interview in the write up?

**Transcription & Recording Commence.**

This is interview X on the date of X.

Transcription and recording has now started, so I will commence the interview. Please note that I will not use your name so not to include on the transcript.

**Section 2- Demographical Characteristics**

1. Basic information: Would you be able to tell me your age, year of the course, and a little bit about your medical school journey?
2. Placement: Can you tell me a little bit about your placement sites this year? Are you currently changing placement sites, or at one site for the entire year?
   a. Do you utilise free accommodation?
3. Living situation: who do you live with?
   a. Aim to ascertain whether the participant lives with other students (and which types of students), parents or others.

**Section 3- Baseline Diet**

Now, we will move on to discuss your baseline diet and your perceptions towards it.

1. Can you tell me a little bit about your views towards your current diet and your views on it?
2. How close is this to your desired diet? We will be referring to your ‘desired diet’ as the diet you would aim to have in an ideal world.

**Section 4- Barriers to diet & methods to reduce these.**

Participant led: 5 main questions.

1. Can you tell me about any barriers that you think you face, with regards to achieving your desired diet balanced diet?
2. Is there anything that has facilitated/ enabled you with achieving your desired diet?
3. Do you think that being a medical student, you experience more barriers than an average person your age? If so, why?
4. Do you think there is anything from a personal level, that you could do, to reduce the dietary barriers that you face? that could help to reduce these barriers?
5. Do you think there is anything the university could do to help reduce these barriers?

**General follow up questions.**

- How does this affect you?
- What are your thoughts on that and why?
- You mention that (X) is a barrier/ enabler. In what way is this so?

**Section 5- Conclusion**

Just before we conclude the study, I want to check the following questions:

1. Is there anything that you feel like you would like to add? Are there any barriers that you do not think we have explored in enough detail?
2. Main barrier: If I had to ask you, what would be the main barrier for you, to achieving your desired diet, what would it be?
3. Main enabler: If I had to ask you, what would be the main enabler for you, to achieving yoru desired diet? from barriers, do you have any specific enablers to a balanced diet?
4. Main strategy: Now, what would be the main strategy or idea that you have, to help reduce these barriers for yourself and for other students?

Thank you very much for taking part in my study. Please do get in touch if you have any further questions.

**Transcription and recording stop**.
